# Mechanochemical Synthesis of Sustainable Ternary and Quaternary Nanostructured Cu_2_SnS_3_, Cu_2_ZnSnS_4_, and Cu_2_ZnSnSe_4_ Chalcogenides for Thermoelectric Applications

**DOI:** 10.3390/nano13020366

**Published:** 2023-01-16

**Authors:** Himanshu Nautiyal, Ketan Lohani, Binayak Mukherjee, Eleonora Isotta, Marcelo Augusto Malagutti, Narges Ataollahi, Ilaria Pallecchi, Marina Putti, Scott T. Misture, Luca Rebuffi, Paolo Scardi

**Affiliations:** 1 Department of Civil, Environmental and Mechanical Engineering, University of Trento, Via Mesiano 77, 38123 Trento, Italy; 2 Consiglio Nazionale delle Ricerche—SuPerconducting and Other INnovative Materials and Devices Institute (CNR-SPIN), Department of Physics, Via Dodecaneso 33, 16146 Genova, Italy; 3 Department of Physics, University of Genova, Via Dodecaneso 33, 16146 Genova, Italy; 4 Department of Materials Science & Engineering, Alfred University, Alfred, NY 14802, USA; 5 Argonne National Laboratory, 9700 South Cass Avenue, Lemont, IL 60439, USA

**Keywords:** Cu-based ternaries and quaternaries, thermoelectricity, Cu_2_ZnSnS_4_, Cu_2_ZnSnSe_4_, Cu_2_SnS_3_, mechanochemistry, order–disorder, X-ray diffraction, density function theory, ab-initio molecular dynamics, Raman spectroscopy

## Abstract

Copper-based chalcogenides have emerged as promising thermoelectric materials due to their high thermoelectric performance, tunable transport properties, earth abundance and low toxicity. We have presented an overview of experimental results and first-principal calculations investigating the thermoelectric properties of various polymorphs of Cu_2_SnS_3_ (CTS), Cu_2_ZnSnS_4_ (CZTS), and Cu_2_ZnSnSe_4_ (CZTSe) synthesized by high-energy reactive mechanical alloying (ball milling). Of particular interest are the disordered polymorphs of these materials, which exhibit phonon-glass–electron-crystal behavior—a decoupling of electron and phonon transport properties. The interplay of cationic disorder and nanostructuring leads to ultra-low thermal conductivities while enhancing electronic transport. These beneficial transport properties are the consequence of a plethora of features, including trap states, anharmonicity, rattling, and conductive surface states, both topologically trivial and non-trivial. Based on experimental results and computational methods, this report aims to elucidate the details of the electronic and lattice transport properties, thereby confirming that the higher thermoelectric (TE) performance of disordered polymorphs is essentially due to their complex crystallographic structures. In addition, we have presented synchrotron X-ray diffraction (SR-XRD) measurements and ab initio molecular dynamics (AIMD) simulations of the root-mean-square displacement (RMSD) in these materials, confirming anharmonicity and bond inhomogeneity for disordered polymorphs.

## 1. Introduction

The depletion of fossil fuels and their harmful effects on the environment have encouraged the scientific community to explore the field of renewable energy. In particular, technologies enabling the harvesting of dispersed sources of energy, such as photovoltaics, thermoelectrics, and piezoelectrics, have attracted great interest [1]. Among them, thermoelectric (TE) technology has been proposed for waste heat recovery, off-grid power generation, as well as refrigerant-free cooling and thermal regulation. Compared to conventional power generation methods, TE materials can directly harness heat, a low-grade form of energy, and convert it into electricity, a high-grade form of energy. The solid-state nature, absence of noise and moving parts, long life span, and portable nature are some advantages of TE generators [2]. Recently, interconnected sensors for various applications, including the Internet of Things, and hybrid thermoelectric–photovoltaic devices have attracted the TE research community and industry [3].

In terms of performance, the maximum energy conversion efficiency of a TE material is determined by the dimensionless figure of merit (*zT*) proposed by Ioffe in 1957 [4], which is defined as

(1) $$zT=\frac{S^{2}\sigma }{\kappa }T=\frac{S^{2}\sigma }{\left(\kappa_{l}+\kappa_{e}\right)}T$$

where *S* is the Seebeck coefficient (thermopower), *σ* is the electrical conductivity, *κ* is the thermal conductivity, and *T* is the absolute temperature. The power factor *S^2^σ* is associated with electrical transport. The thermal conductivity comprises two parts, the lattice part *κ_l_* and the electronic part *κ_e_*. To achieve a high *zT*, a large *S* is required to ensure a high output voltage, a high *σ* to reduce Joule heat losses, and a low *κ* to maintain the temperature gradient between the hot and cold sides. However, these three transport parameters, *S*, *σ*, and *κ*, are highly interdependent and depend on the band structure, carrier concentration, microstructure, and many other factors. The following three equations show the linkage of the transport parameters,

(2) $$S=\frac{8\pi^{2}k_{B}^{2}}{3eh^{2}}m^{*}{\left(\frac{\pi }{3n}\right)}^{2/3}T$$

(3) σ=neμ

(4) $$\kappa_{e}=L\sigma T$$

where *k_B_*, *h*, m*, *n*, *μ*, *e*, and *L* represent the Boltzmann constant, the Planck constant, the effective mass of charge carriers, carrier concentration, carrier mobility, the charge of an electron, and the Lorenz number, respectively [5].

Equation(1) shows that *κ_l_* is the only independent variable in transport properties. Therefore, reducing it is among the most effective ways to increase *zT*. There are several strategies to decrease the lattice thermal conductivity, such as introducing rattling atoms [6], nanostructuring, lattice anharmonicity that can be induced by layered structures [7], or lone pairs [8], and introduction of disorder in the crystal structure [9].

An optimal range for carrier concentration (10^18^–10^20^ cm^−3^) and low thermal conductivity are desired for high-performing TE materials [5]. To this end, state-of-the-art TE materials employ heavy-mass elements, such as Pb, Hg, Cd, Te, Sb, and Bi [10,11,12], materials which are often rare and/or toxic. For the sake of scalable commercial application, intensive efforts are required to explore non-toxic, earth-abundant, environmentally friendly, sustainable, and economically viable materials. Cu-based sulfides/selenides have recently attracted much attention in TE research, in part because of their earth abundance. Devices employing these materials showed power output per unit cost comparable to high-performance thermoelectric devices, underlining the relevance of obtaining a thorough understanding of the transport mechanism in these materials [13,14]. Mechanochemical syntheses via high-energy reactive milling offer the unique advantage of producing various disordered polymorphs of these compounds. This single-step and solvent-free method can alloy materials with different melting points, such as transition metals and chalcogenides, due to the highly entropic environment of the milling [15,16,17,18]. Such an environment also favors the disordering of these Cu-alloys. Recently, we have synthesized and stabilized various polymorphs of Cu_2_SnS_3_ (CTS) [9], Cu_2_ZnSnS_4_ (CZTS) [19], and Cu_2_ZnSnSe_4_ (CZTSe) [20] through this bottom-up ball-milling technique. 

The constituting elements of ternary CTS are earth abundance and eco-friendly, making it suitable for sustainable and large-scale use. Researchers have investigated CTS polymorphs for a number of applications, such as optoelectronics [21], sensors [22], absorber layer of solar cell [23], and TE [24]. The complex crystallographic structure of CTS allows to synergistically tune the electronic and thermal transport properties, resulting in a high thermoelectric figure of merit. Heavily In [24] and Zn [25] doped CTS systems were among the first CTS systems investigated for TE application in the last decade. Subsequently, several other cationic substitutions were investigated with the aim of improving the TE performance by tuning the carrier concentration and introducing structural disorder such as Co [26], Cu [27], Ni [28], Fe [29], and Mn [30]. Recently, Wei et al. [31] showed effective suppression of thermal conductivity of CTS by manipulating the phase composition and twin boundary engineering. Zhao et al. achieved the highest *zT*∼0.9 above 700 K by simultaneous doping with cobalt and antimony [32]. Moreover, 3D modulation doping has also been studied in nanocomposites CTS, which have an ultra-low thermal conductivity and high *zT* [33]. The disordered cubic phase of CTS/CTSe was also synthesized without external doping [9,34,35,36].

The quaternary chalcogenides CZTS and CZTSe have also been investigated for application in other fields of energy materials. In particular owing to their structural similarity with Cu_2_InGaSe_4_ (CIGS), they have been extensively studied as a sustainable replacement for the absorber layer in thin-film solar cells due to an optimal, direct band gaps and high absorption coefficients [37,38]. CZTS has also been explored as a hole transport layer in perovskites solar cells [39]. In addition, both CZTS and CZTSe have received attention for thermoelectric application [40]. When the carrier density is optimized, they have shown potentially outstanding mid-to-high temperature performance, in part due to their complex structure and intrinsically low thermal conductivity. This was achieved through Cu-rich [40,41,42,43] or Sn-deficient [44,45] stoichiometries. These strategies rely on the conversion of the insulating ZnS_4_/Se_4_ and SnS_4_/Se_4_ pathways into conducting pathways by the substitution of Zn and Sn for Cu in the CZTS/Se compounds [40]. The vacancy cluster-induced localized disorder on the domains reported for CZTSe by Li et al. [45] also improved the TE performance for this system, an effect which proved to be superior to the enhancement due to phonon-glass electron-crystal from nanostructuring. The extrinsic doping with Na [46], Ag [47], Ni [48], and Ga [49] have also shown significant improvement in the performance of these materials. In addition, the disorder in these systems introduced by temperature [50,51], chemistry [45], or synthesis method [20,52,53] revealed to be critical to their improvement as TE materials.

In the present work, we summarize recent advances in disordered CTS, CZTS and CZTSe materials and discuss the effects of cation disorder on the TE properties. The rest of this article is structured as follows. First, we examine the various crystallographic structures of these compounds. Subsequently, we discuss the intrinsic disorder present in these systems, as reflected in the root-mean-square displacement (RSMD), through a combination of ab initio molecular dynamics (AIMD) and in situ SR-XRD. This is followed by a discussion of the rich and diverse phenomenology behind the electron and phonon transport mechanisms in these disordered materials, and how they affect the key thermoelectric properties. Finally, we conclude with a brief description of the atypically inverse dependence of the electrical conductivity on the sample grain size in both CZTS and CTS, and the different origins of this behavior in the two compounds.

## 2. Structure and Nature of Disorder

Various preparation methods, such as solid-state reaction [28], hot injection [54], and ball milling [50,51], have been proposed in the literature for the synthesis of Cu-based chalcogenides [53,55]. They have a low formation energy, which makes them suitable to be prepared via high-energy reactive milling in a highly controlled atmosphere [56]. Usually, the disordered phases of CTS are prepared by high-temperature solid-state reactions with acceptor doping [25,31], while the disordered cubic phases of CZTS and CZTSe coexist in small phase fractions with their ordered phase. In our previous work, the disordered phases of all three compounds were stabilized by high-energy reactive milling [9,34,35,50,52]. This is only possible because of the highly entropic environment promoted by ball milling, which is known to form unique metastable phases. The modern understanding of the mechanochemical reaction is based on the transfer of electrons and exchange of ions [15] in the interfacial component of the milled material during the process of phase nucleation. Due to this phase formation process, a more “kinetic” state of matter is achieved when compared to other solvent-dependent bottom-up approaches [18,57]. This characteristic of mechanochemistry promotes the formation of metastable phases and, for the systems studied here, the structural disorder attained is so high that the crystal structure is better described by a cubic symmetry. The preparation of the samples by ball milling also offers the unique advantage of preserving the nanostructure, maintaining a high density of crystalline defects, and an inhomogeneous distribution of finely dispersed crystalline grains. Apart from this, mechanochemistry is in line with the 12 principles of green chemistry [58], especially in terms of limited solvent use, lower energy consumption, and suitability for low-cost large-scale production [53].

Regarding the Cu-based alloys, CTS has three widely reported structures—the ordered monoclinic (*Cc*), tetragonal (*I*$\bar{4}$*2m*) and the disordered cubic (*F*$\bar{4}3$*m*). Recently, an orthorhombic (*CmC2_1_*) CTS polymorph was produced [59]. CZTS and CZTSe on the other hand, have three polymorphs: an ordered tetragonal (*I*$\bar{4}$), a disordered tetragonal (*I*$\bar{4}$*2m*) with Cu-Zn disorder, and a disordered cubic (*F*$\bar{4}3$*m*) with a full-cation disorder. The schematic diagram of the structures, all based on a diamond-like tetrahedral coordination, is presented in Table 1. The disordered cubic polymorphs are a variant of the zincblende (ZnS) structure, which consists of tetrahedral S-cages (Wyckoff position 4*c*) with a cation (Zn) positioned in the center (4*a*). In disordered CTS, the Zn cation is randomly replaced by Cu and Sn, leading to a partial cation occupation of 2/3 and 1/3 for Cu and Sn, respectively. In the case of the disordered cubic CZTS and CZTSe, the Zn cation is replaced by Cu, Zn and Sn in partial occupation of 1/2, 1/4, and 1/4, respectively. 

CZTS and CZTSe show a reversible temperature-dependent phase transition, also observed in analogous quaternary chalcogenides [60]. In the ordered form, they crystallize in the Kesterite structure with the tetragonal space group *I-4*, consisting of alternating Cu-Zn and Cu-Sn layers sandwiched between the anion (S/Se) layers. At relatively low temperatures, CZTS/Se shows a structure transition from *I-4* to a partially disordered tetragonal structure with space group *I-42m*. In this, due to the large chemical and size mismatch between Cu and Sn, an appreciable disorder is only possible on the Cu–Zn sublattice, while the Cu-Sn layer remains ordered. Nevertheless, the structural complexity of these quaternary chalcogenides frequently causes the occurrence of various types of structural defects even close to room temperature. The most common one is the Cu_Zn_ substitutional, which is believed, along with Cu vacancies, to be the cause of the *p*-type nature of Kesterite and has the lowest formation energy among the possible defects [61,62,63,64]. 

**Figure 1:**
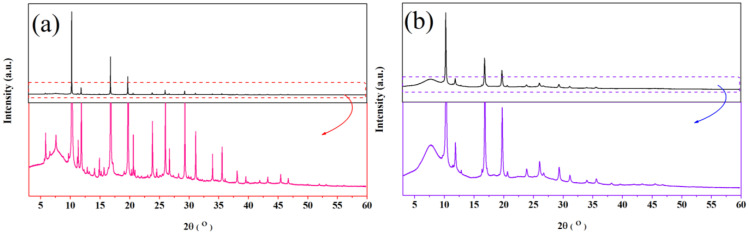
SR-XRD measurements on monoclinic (**a**), and cubic (**b**) Cu_2_SnS_3_ polymorphs collected at 350 °C. The zoomed images show the superstructure Bragg peaks (2θ < 10°) for monoclinic CTS phase representing (110), (11-1), and (021) reflections. These superstructure Bragg peaks are absent for the cubic CTS phase. The broad peak with diffused background at low 2-theta is due to the use of borosilicate glass for the dilution of samples.

The difference between monoclinic and cubic polymorphs of CTS is quite evident from the structural data. It has been shown by the combination of XRD, SAED (see Supplementary Figure S2) and Raman spectroscopy [9]. The cubic CTS did not show critical low-angle reflections in XRD and SAED patterns. Moreover, two distinctive Raman modes were observed (at 283 cm^−1^ and 337 cm^−1^) for disordered cubic CTS [9]. Regarding the CZTS/Se system, the difference between disordered cubic CZTS/Se (*F-43m*) and disordered tetragonal CZTS/Se (*I-42m*) are the superstructure reflections visible through the low-angle Bragg-peaks in XRD and the observed new rings in the SAED patterns [45,46,47]. These transitions were also observed in DSC measurements for CZTS/Se [20,52]. The SR-XRD for the monoclinic and cubic CTS at 350 °C is shown in Figure 1. The SR-XRD result for the different temperature is shown in the Supplementary Materials (Figure S1). Table 2 reports the structural parameters and band gap of various polymorphs.

### Role of Disorder

The combination of molecular dynamics simulations and XRD measurements with WPPM modelling [70,71] has been demonstrated to be a simple yet effective measure of atomistic disorder [66], with a remarkable agreement between theory and experiment. To observe the effect of structural disorder on the RMSD, the temperature evolution of the ordered and disordered CTS, CZTS [66], and CZTSe was investigated by temperature-dependent XRD and AIMD simulations. The disordered CTS presented a higher value of the Grüneisen parameter (a measure of anharmonicity) when investigated by nuclear inelastic scattering, results which were verified by calculating the mode Grüneisen parameter in the quasi-harmonic approximation using DFT [34]. As such, the evolution of RMSD value was expected to be higher in the case of disordered CTS/CZTS/Se as compared with the ordered counterpart. XRD patterns for CTS were collected in the temperature range from 323 to 773 K with a step size of 50 K. While for CZTSe were collected from 25 to 300 K. In Rietveld refinements, the value of isotropic Debye–Waller coefficient (*B_iso_*) for all the cation positions was assumed to be the same. From the *B_iso_*, the RMSD was calculated using the following relation, where $\left\langle u^{2}\right\rangle$ is the mean squared displacement (MSD):

(5) $$B_{iso}=\frac{8\pi^{2}}{3}\langle u^{2}\rangle$$

**Figure 2:**
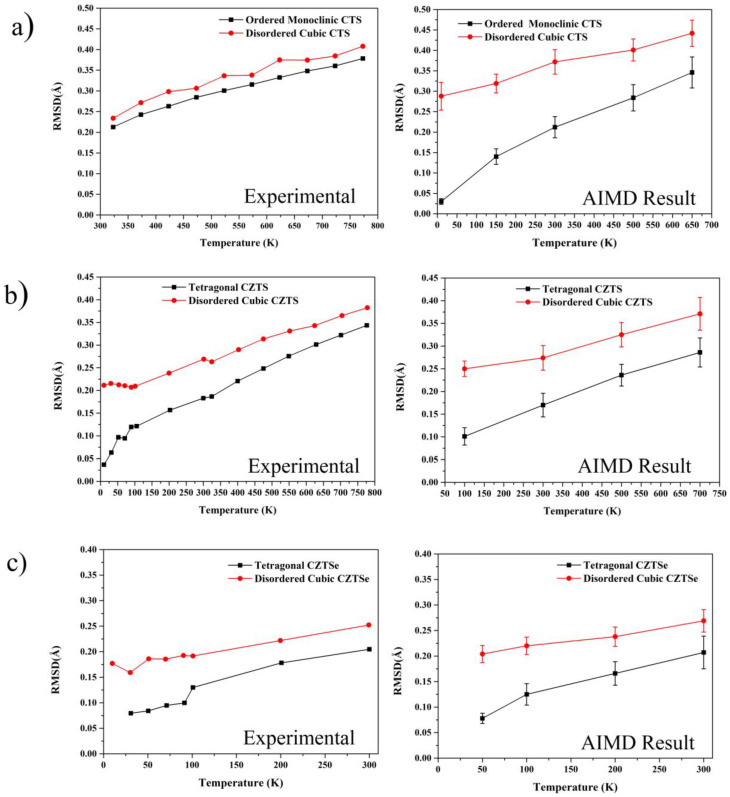
Temperature variation of RMSD calculated from SXRD and AIMD simulation for CTS (**a**), CZTS (**b**), and CZTSe (**c**).

Figure 2 shows the results of RMSD calculated from the temperature-dependent XRD and AIMD simulations. As expected, the value of RMSD increased with temperature for all the polymorphs, implying that the amplitude of the atomic vibrations increases with temperature. The RMSD value for the disordered polymorph was higher than the ordered phase, pointing to an increased static disorder. This corresponds to the static (temperature-independent) distortion in the crystalline lattice due to cation disorder and is connected to higher anharmonicity and bonding inhomogeneity in the disordered phase, which is then directly responsible for the ultra-low thermal conductivity. For CZTS/Se, the RMSD values calculated using AIMD simulations agree well with the experimental results. 

For CTS, the RMSD values calculated from the experiment and the AIMD simulation are in qualitative agreement. A slight deviation was observed in the absolute RMSD value was observed. The reason for this discrepancy could be due to the clustering effect observed in the CTS systems [72]. The clustering effect in CTS structures restricts the formation of S−Cu_4_, S−CuSn_3_, and S−Sn_4_ motifs. In ordered CTS, a regular distribution of S−Cu_2_Sn_2_ and S−Cu_3_Sn motifs was observed, while in disorder CTS, S−Cu_2_Sn_2_ motifs form nanometer-scale clusters. This consideration of clustering was not considered in the modelling of the system, due to the limitation of plain wave density functional theory in handling systems of the nanometer range.

**Figure 3:**
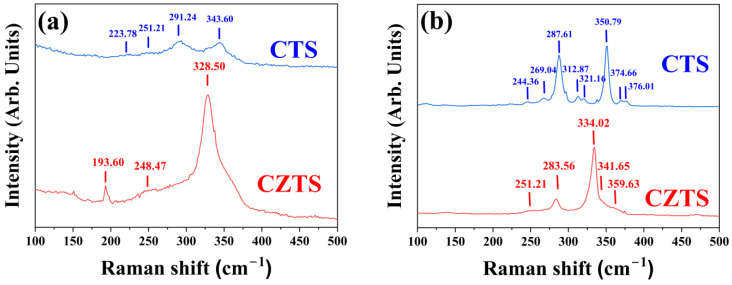
Raman spectra collected with a 488 nm laser on as-milled (**a**) and thermally treated (**b**) samples of CTS and CZTS. The samples in (**a**), in the disordered cubic phase, display significantly broader peaks than the ordered (monoclinic CTS and tetragonal CZTS) phases in (**b**). This is believed originating from cation disorder itself, likely causing variable coordination environments and inhomogeneous bonds, leading to a spread in vibrational modes.

Overall, the emerging key result is that the disordered compounds present an additional temperature-independent contribution to the RMSD. Indeed, the highly varying coordination environment and bonding inhomogeneity in these phases likely lead to non-uniform distortions in the coordination tetrahedra. This, by bulk-averaging techniques such as XRD, is collectively observed as an increase in the RMSD. This interpretation is also supported by the significant broadening of Raman peaks for the disordered compared to the ordered compounds, illustrated for CZTS and CTS in Figure 3. As visible, the disordered phases show peaks approximately at the same locations, but significantly broader. This likely reflects the larger distribution of vibrational frequencies associated with specific phonon modes, due to the contribution of highly inhomogeneous bonds and non-uniform coordination environments. A similar broadening was also observed by other studies on mechanically alloyed CZTS in the literature [73].

## 3. Electronic Properties

### 3.1. Seebeck Coefficient, Resistivity, and Power Factor

**Figure 4:**
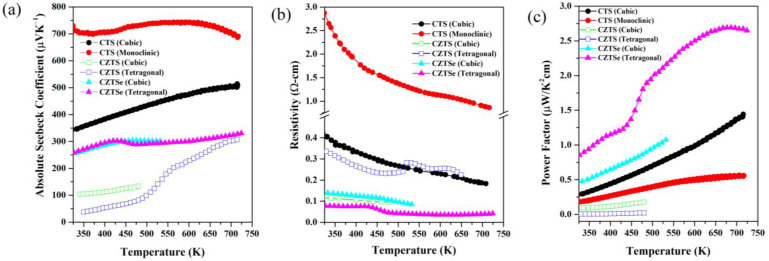
Temperature-dependent Seebeck coefficient (**a**), electrical resistivity (**b**), and power factor (**c**) curves for CTS, CZTS, and CZTSe polymorphs retrieved from [20,34,51,52].

All polymorphs of CTS, CZTS and CZTSe exhibit a positive absolute Seebeck coefficient, due to their p-type nature (shown in Figure 4). Both the ordered and disordered polymorphs of CTS are stable up to 723 K. The tetragonally ordered CZTS/Se samples were also studied up to 723 K and showed a reversible phase transition to the tetragonally disordered phase above 500 and 400 K, respectively. However, the cubic/disordered polymorphs of CZTS and CZTSe from mechanical alloying were stable up to ~500 and 600 K, respectively.

As all the samples discussed were undoped, they exhibited a non-degenerate nature of the electrical resistivity. Monoclinic CTS presents the highest Seebeck coefficient (S~ 700–750 µV/K) and a non-linear trend in temperature. It has been reported that this trend is caused by bipolar contributions to charge carriers [25]. Interestingly, tetragonal CZTS exhibits a sharp increase in the Seebeck coefficient due to the order–disorder transition, while the same transition causes a slight decrease in the Seebeck coefficient for CZTSe. The origin of this behavior is explained in the following section. It is important to note that CZTSe samples are generally more conductive than CTS and CZTS, with tetragonal CZTSe having the lowest resistivity (< 0.1 Ω-cm) among these samples. This is due to the Cu-Se conduction network, which should be more conductive than the Cu-S conduction network. 

In contrast with ordered CTS, disordered CTS shows a linearly increasing Seebeck coefficient. Furthermore, disordered CTS presents a lower Seebeck coefficient and resistivity than monoclinic/ordered CTS. 

In terms of overall power factor, CZTSe samples perform better than CZTS and CTS. When comparing polymorphs of the same composition, it can be generalized that the disordered ones (i.e., disordered cubic for CTS and CZTS and disordered tetragonal CZTSe) have better performance than their ordered counterparts.

### 3.2. The Role of Electronic Structure

As discussed, the tetragonal order–disorder transition in CZTS/CZTSe manifests in a variation of the Seebeck value, which sharply increases for CZTS, and moderately decreases for CZTSe at the transition temperatures of ~500 and ~450 K, respectively. This behavior is associated with two different mechanisms, as shown by first-principles calculations [20,51]. In both cases, the disorder contributes to a flattening of the bands (increasing the Seebeck coefficient) and suppression of the band gap (decreasing the resistivity). However, in the case of the sulfide, the Cu-Zn disorder generally leads to a convergence in the energy of the three valence bands at the Gamma point, whereas it leads to a divergence of the same bands in the case of the selenide. This opposite behavior is the result of two competing effects: homogenization of the charge density and crystal field splitting. In CZTS, the Cu-Zn disorder leads to a homogenization of the local bonding environment around each ion since the electronic configurations of Cu and Zn differ by only a single electron. In contrast, the presence of the heavier Se ions in CZTSe leads to a stronger crystal field splitting, causing the energy levels to diverge. Despite this variation in mechanism, both disordered tetragonal polymorphs show an improved power factor compared to their ordered counterparts. The conduction and valence bands are derived from anion-*p* and Cu-*d* electrons. For the cubic polymorphs of CZTS and CZTSe, the bulk band structures are significantly flattened compared to the ordered polymorphs, corresponding to a reduced charge carrier mobility, in turn resulting in an increased Seebeck coefficient. In addition, the band extrema of the two quaternary chalcogenides show deviations from the standard parabolic nature, in the form of anti-crossing or “camel’s-back” features (see Figure 10a). These features are accompanied by band inversion—a localized reversal of orbital contributions—with Cu-*d* orbitals dominating the conducting band minimum (CBM) and anion-*p* orbitals dominating the valence band maximum (VBM). Band inversion is associated with topological insulators, and in this respect, disordered cubic CZTS is of particular importance, as these topological features are not present in either the ordered or disordered tetragonal forms. This indicates a topologically non-trivial behavior caused by the complete disorder of the cations, and as such, it has been predicted to be the first topological Anderson insulator in a real material system [74]. In CZTSe, on the other hand, all polymorphs show band inversion in the bulk band structure and have been theoretically predicted to be topological insulators. 

**Figure 5:**
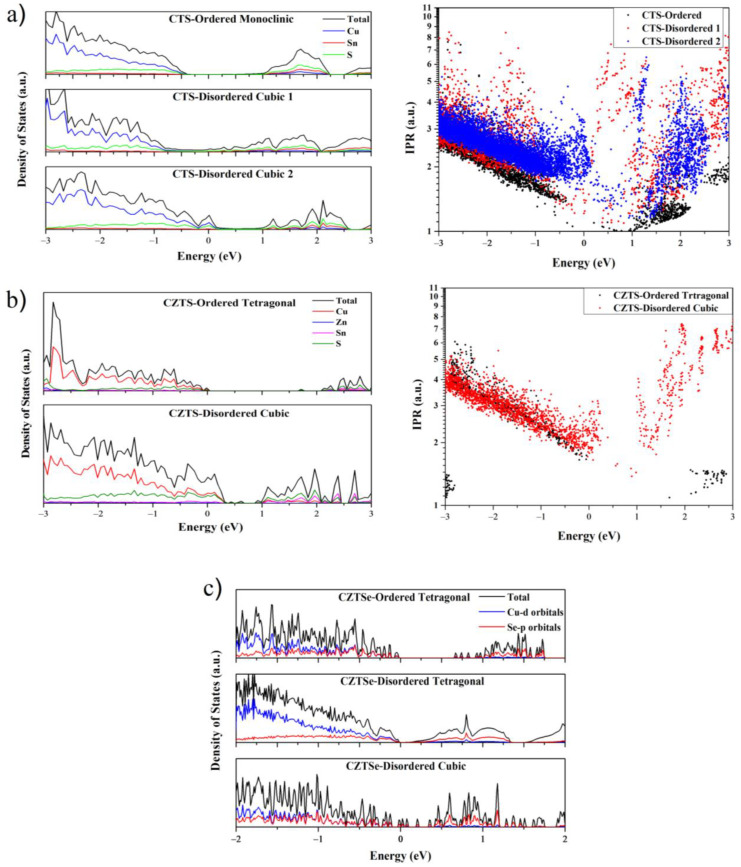
The total and atomic projected electronic density of states for CTS ordered monoclinic, CTS Disordered Cubic 1 (Cu-21, Sn-11, S-32), and CTS Disordered Cubic 2 (Cu-22, Sn-10, S-32) (**a**), CZTS (**b**), and CZTSe (**c**) polymorphs. Here, the Fermi level is set to zero. The IPR for the CTS and CZTS shows localized states near the Fermi level.

**Figure 6:**
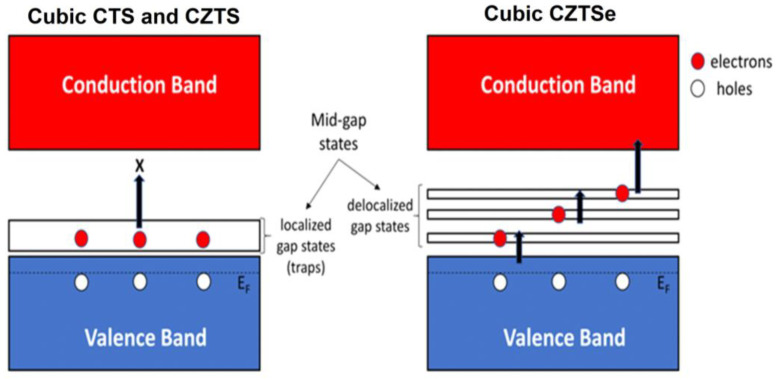
A schematic diagram showing the effect of mid-gap states: In cubic CTS/CZTS, the localized nature of these states acts to unilaterally enhance hole concentration at finite temperature by ‘trapping’ electrons. For cubic CZTSe instead, these states are delocalized within the gap, and therefore do not act as traps.

The electronic density of states of the different polymorphs of the three compounds shown in Figure 5 shows strong similarities. In the VBM of CTS, the main contribution to DOS is made by Cu *3d* and S *3p* orbitals, while the CBM consists of Sn *5s* and S *3p* orbitals. In CZTS, the states in the VBM are dominated by Cu *3d* electrons, while the states in the CBM consist mainly of S *3p* orbitals. In the case of CZTSe, the valence band in each polymorph is strongly hybridized between contributions from Cu *3d* and Se *4p* orbitals, while the conduction band is dominated mainly by Se *4p* orbitals. The structural disorder leads to fluctuations in the potential energy landscape of the lattice, allowing energy levels within the forbidden gap, which are responsible for the band tailing and reduced band gap. In the case of CZTS and CTS, these states are highly localized and located near the valence band, can be observed from the inverse participation ratio (IPR) plot in Figure 5. These states trap electrons from the VB, leading to an increase in the number of holes without a corresponding increase in the number of electrons in the conduction band. This leads to an increase in the *p*-type charge carrier concentration, as measured experimentally. Figure 6 shows a schematic diagram of the effects of highly localized intermediate gap states near the valence band. These localized trap states lead to a significantly higher power factor for disordered cubic CZTS and CTS. However, in the case of CZTSe, these states are distributed over the entire gap and are less localized. Therefore, the cubic polymorph shows a conventional behavior with a lower conductivity and a higher Seebeck coefficient, with an overall lower PF comparable to the tetragonal phase.

The electronic structure calculations presented in the previous works were limited to the use of the Perdew–Burke–Ernzerhof (PBE) [75] exchange-correlation function due to the limited computational resources and time constraints. In this work, the electronic structure calculations were performed using the hybrid Heyd–Scuseria–Ernzerhof (HSE) [76] exchange-correlation function, which provides a better description of the band gap. These results confirm that band tailing and mid-gap states are an intrinsic part of the electronic density of states for the disordered structures.

## 4. Thermal Conductivity

**Figure 7:**
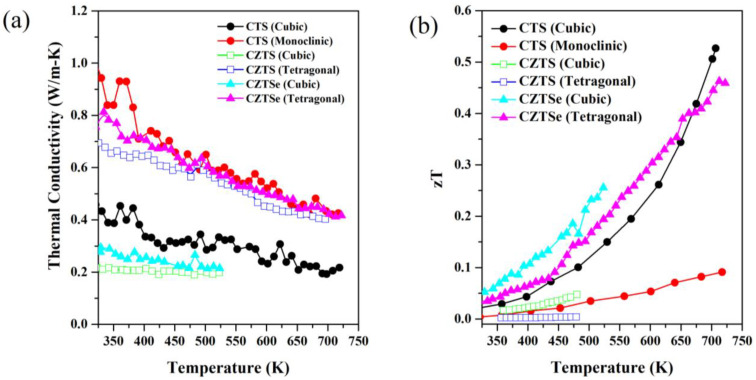
Temperature-dependent thermal conductivity (**a**) and *zT* (**b**) for CTS, CZTS, and CZTSe polymorphs data retrieved from [20,34,51,52].

The most dramatic improvement in the TE properties of these disordered cubic chalcogenides is due to a significant reduction in thermal conductivity. Figure 7a shows the variation of thermal conductivity of all polymorphs. The thermal conductivity for CTS and tetragonal phases of CZTS/Se follows a *T^−1^* trend, indicating that the thermal conduction is dominated by Umklapp scattering. In the case of cubic CZTS/Se, the low values of thermal conductivity remained almost constant over a wide range of temperatures. This suggests an additional, temperature-independent contribution to the low thermal conductivity. The trend in thermal conductivity clearly shows the effects of structural disorder. It is worth noting that all the samples discussed have a lower density (~20–25%) than the theoretical one, which is due to the preparation method where manual cold pressing was used. However, the comparison between the above samples is fair as they have similar densities and the same preparation method. It should be noted here that the literature increasingly recommends the use of low-density materials for TE applications to reduce the cost and quantity of materials used [77,78]. However, the effect of low density appears to be less pronounced for electronic transport [56]. The ultra-low thermal conductivity of CTS samples was pertinent even after employing spark plasma sintering [35].

Regardless of the three different materials discussed in this article, the disordered cubic polymorphs have the lowest thermal conductivity, featuring values less than half that of their monoclinic and tetragonal counterparts. These samples showed a non-degenerate trend of resistivity as a function of temperature, with high values of resistivity. This suggests that the electronic contribution to κ is minimal and that the lattice thermal conductivity dominates κ.

**Figure 8:**
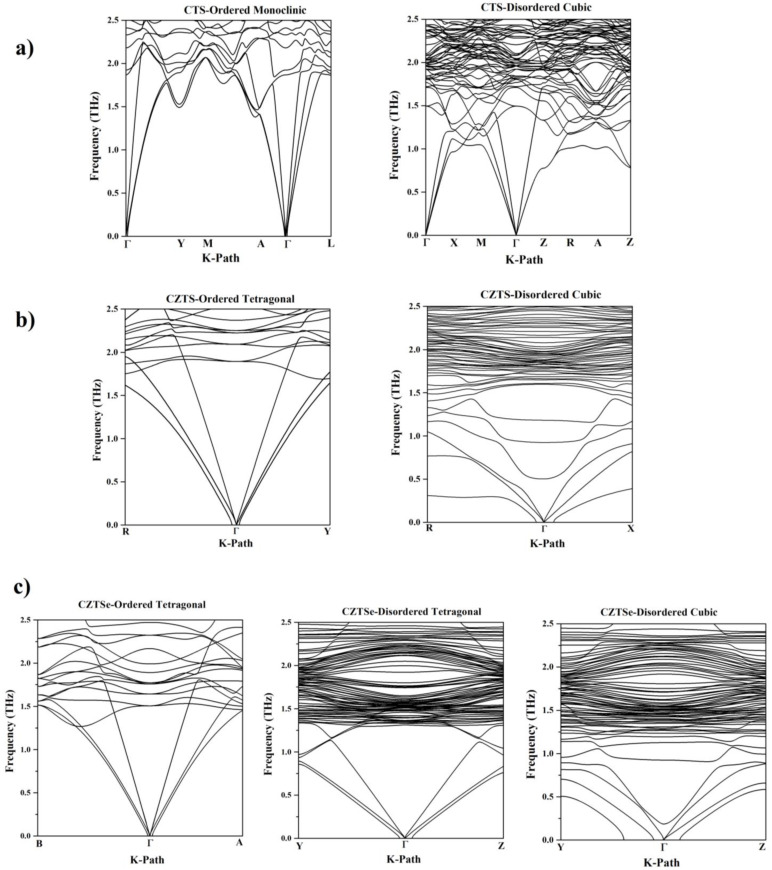
Phonon dispersion curve for CTS (**a**), CZTS (**b**), and CZTSe (**c**) polymorphs. The effect of structural disorder on the phonon dispersion curve manifests itself in the form of a gentle slope of the acoustic branches and the low-lying optical modes.

**Figure 9:**
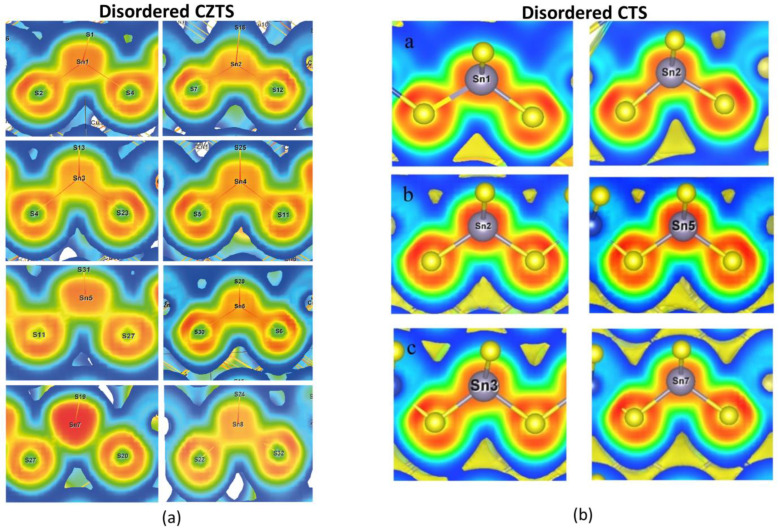
The calculated electron localization function mapped on to the Sn-S bonds in (**a**) disordered CZTS and (**b**) disordered CTS. The ELF distribution for certain Sn ions shows a strong bonding inhomogeneity in both systems, with only CZTS showing lone-pair localization in a fraction of ions (for example see Sn7 in the panel in the bottom left corner of the figure). These images are taken from [34,52].

**Figure 10:**
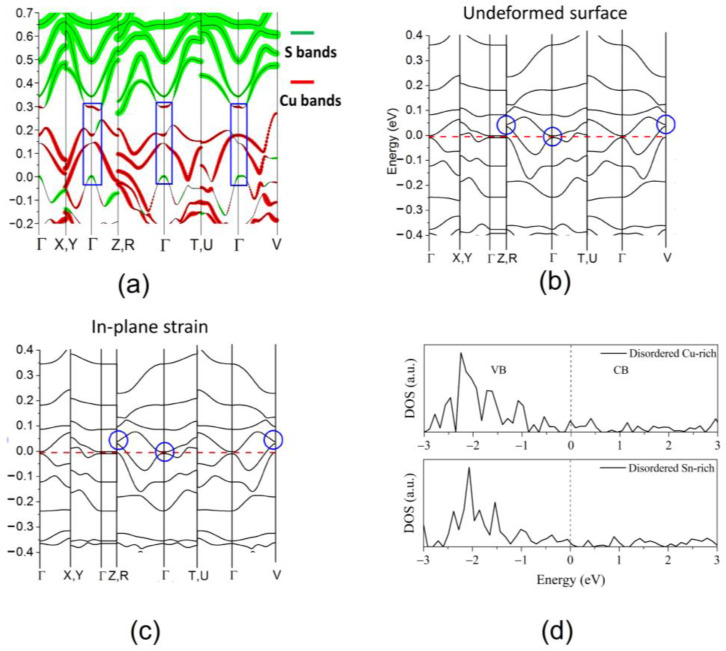
(**a**) Bulk band inversion and (**b**) topological surface states in pristine form and (**c**) under in-plane strain for disordered cubic CZTS; (**d**) surface DOS for disordered cubic CTS showing metal-like surface states. These band structure and density of states plots are taken from [56,74].

The first-principle calculations show that all three cubic compounds have similarities in their phonon dispersion, with a lower slope of the acoustic modes (lower group velocity) compared to their ordered counterparts and low-lying optical modes (Figure 8). These low-lying optical modes interact with the heat-carrying acoustic modes and lead to the dissipation of thermal energy. The possibility of this dissipative scattering is enhanced by the dense band of optical modes present (due to disorder) in all disordered polymorphs above 1.25 THz, which provides multiple channels for scattering. In cubic CZTS, these low-lying modes are directly associated with the rattling of certain Sn ions, which are characteristic features of this compound. Due to the retention of the lone Sn *s2* pair in cubic CZTS, certain Sn ions form electron-poor bonds with the coordinating S ions (see Figure 9a). These weakly bound Sn ions contribute to low-frequency vibrational modes in the DOS vibration and are additionally responsible for the nature of the electronic states within the band gap, which fundamentally affects the electrical transport properties as described above. Interestingly, these rattling ions are not present in CZTSe and CTS. In particular, the electron localization function showed an inhomogeneity in bonding for cubic CTS (see Figure 9b), leading to a higher anharmonicity, which was confirmed by the calculation of the mode Grüneisen parameter by DFT and Nuclear inelastic scattering (NIS) measurements. The strong anharmonicity leads to an overall ultra-low thermal conductivity [34]. 

The disordered cubic polymorphs have a higher *zT* than the monoclinic or tetragonal ones. The inherently low thermal conductivity of high symmetric cubic polymorphs suggests a phonon-glass electron crystal behavior, as the remarkable kappa suppression does not negatively affect the electronic conductivity. Similar effects are also reported for CTS samples prepared by different preparation techniques such as open die pressing(ODP) and traditional sintering, resulting in samples with different proportions of porosity [56].

From near room temperature to intermediate temperatures, CZTSe polymorphs appear to have the best performance among these samples. Cubic CZTSe, which is stable up to 500 K, shows the best performance over the entire measured temperature range, exhibiting a *zT*~0.25 at 500 K. Interestingly, cubic CTS performs better than all other samples at the highest measured temperature of ~723 K, resulting in a *zT*~0.5. This performance is followed by tetragonal CZTSe, which has a *zT*~0.45 at 723 K. Similarly, cubic CZTS performs better than its tetragonal polymorph, which exhibits a *zT*~0.05 at 500 K. The main reason for the low performance of monoclinic CTS and tetragonal CZTS is their higher electrical resistivity and thermal conductivity compared to their disordered phase. Although the overall thermoelectric figure of merit of these materials is moderate, it is worth noting that disorder causes a significant enhancement of *zT*, leading to the highest performance for undoped compositions. This indicates a clear direction for optimization of this family of compounds. Here, we put forward, that the low-density doped samples of these sustainable materials could be interesting study cases for thermoelectric performance analysis, thanks to their highly suppressed thermal conductivity and relatively high electrical conductivity.

## 5. Grain Size Effect on the Transport Properties

The synthesis route of high-energy reactive ball milling helps to stabilize the disordered phases at low temperatures through nanostructuring. The use of different sintering conditions leads to a variation in the average grain size of the nanoparticles. Larger grains have fewer grain boundaries and thus less electron scattering, which usually leads to higher electrical conductivity. Interestingly, disordered cubic CZTS and CTS show the opposite trend, where samples with smaller grain sizes possess higher electrical conductivity. This trend is in contrast to the mechanism of grain boundary scattering normally observed in TE materials [79]. In fact, the evidence points to beneficial surface effects in these compounds. Interestingly, the reason for this trend is quite different for the two compounds—the small-grained samples (below 50 nm) [35] from CTS show an increase in carrier concentration compared to the larger-grained samples, while CZTS shows an increase in carrier mobility.

The topological properties of cubic CZTS should favor large surface mobility. This should partially compensate for the low bulk mobility observed instead in the band structure, which is related to bulk cation disorder. To highlight the contribution of the surface to mobility, one can look at the behavior of samples where surface states should be increasingly dominant (e.g., the trend of mobility with progressively smaller grain size; see [74]). Alternatively, measurements at low temperature or in the presence of a magnetic field should highlight the contribution of surface states. Indeed, the positive contribution to magnetoresistance at low temperature is greater in the cubic polymorph than in the tetragonal phase (see Supplementary Figure S3), indicating greater carrier mobility in the former sample. The mobility measurements below room temperature show an order of magnitude higher upper limit for the cubic phase compared to the tetragonal phase (see Supplementary Figure S4). The greater mobility of the cubic polymorph below room temperature (where elastic scattering due to disorder should dominate over inelastic scattering) is experimental evidence in favor of the topological surface states hosted in cubic CZTS due to the theoretically predicted topological Anderson insulator (TAI) behavior [74]. These topologically protected, quasi-gapless states, immune to backscattering, are then responsible for the transport of charge carriers with higher mobility, leading to increased electrical conductivity.

For CTS, the improvement in charge carrier concentration is attributed to the stoichiometry of the grain surface, which forms incomplete bonds due to the change in coordination number caused by surface termination. Due to the dangling bonds on the surface, localized states near the Fermi level are present. These dangling bonds provide additional charge carriers (holes), which increase the charge carrier concentration and lead to degenerate semiconductor-like behavior [35]. Similar results have been obtained for the disordered CTSe system [80]. 

## 6. Conclusions

Herein, we have presented a comparative study of the TE properties for various disordered polymorphs of the low-toxicity, earth-abundant chalcogenides CTS, CZTS, and CZTSe, synthesized via high-energy reactive milling. The low-cost synthesis route is crucial in stabilizing the polymorph with cubic structures and complete cation disorder. The CTS system exhibits a monoclinic and a disordered cubic arrangement, while CZTS and CZTSe show tetragonal, disordered tetragonal, and disordered cubic structures. The effect of disorder was quantified by calculating the RMSD for ordered and disordered phases using AIMD simulations and comparing the results with temperature-dependent XRD. This structural disorder promotes the optimization of the electrical and thermal transport properties through various mechanisms. The ternary disordered CTS presents a better electrical conductivity than the ordered CTS due to the reduction in band gap and band tails, while the large anharmonicity and inhomogeneous bonds lead to extremely low thermal conductivity. The quaternary systems of CZTS and CZTSe show a temperature-activated phase transformation for the tetragonal phase from an ordered tetragonal to a partially disordered tetragonal arrangement. Moreover, CZTS and CZTSe can be stabilized at low temperatures in a novel disordered cubic phase with full cation disorder. This disorder manifests itself in the form of simultaneous enhancement of the Seebeck coefficient and electrical conductivity in CZTS due to band engineering. In addition, disordered cubic CZTS has been predicted to be a topological Anderson insulator, with topologically protected surface states, a prediction supported by the experimental measurements of high mobility at very low temperatures. Furthermore, because of the bonding inhomogeneity, the disordered cubic phases of the two quaternary compounds also showed ultra-low thermal conductivity, due to anharmonicity, as well as low phonon group velocity and low-lying optical modes. The RMSD showed a higher value for disordered phases, confirming a higher degree of anharmonicity in the cubic disordered phases, which is responsible for ultra-low thermal conductivity. Finally, both CTS and CZTS show the presence of beneficial surface states, making these materials highly suitable for nanostructuring. 

The combination of these diverse and intriguing transport mechanisms and the high chemical tunability of the complex structures leads to competitive thermoelectric performance and makes this class of materials a promising candidate for the fabrication of high-performance, sustainable thermoelectric devices.

## 7. Materials and Methods

**Synchrotron:** High-resolution synchrotron radiation X-ray diffraction (SRXRD) measurements were performed at the 11bm beamline of the Argonne National Laboratory. Data were collected at 300, 200, and 100 K using a blower for cooling and at a wavelength of 0.45820 Å, in the 2θ range 0–50°. In the temperature range 90–10 K, data were collected every 20 K using a Helium cryostat for cooling, a wavelength of 0.44262 Å and a 2θ range of 0–46°. LaB_6_ standard patterns were collected to model the instrumental profile. Specimens were loaded in an Ar filled glovebox in 0.3 mm-diameter (for CZTSe) and 0.5 mm-diameter (for CZTS) Kapton or glass capillaries and diluted with 70%vol borosilicate glass powder. This was done to reach linear absorption coefficients µR < 0.3 and keep systematic deviations in intensity between low and high angles below <1%, thus yielding greater confidence in the estimation of Debye–Waller coefficients.

In a similar manner, temperature-dependent SXRD measurements were performed on ordered and disordered CTS samples from 50 to 500 °C with a step of 50 °C at MS-Xo4SA: Materials Science beamline, Swiss Light Source, Paul Scherrer Institut. SXRD data were collected using **MYTHEN II** detector in 2θ range 0–60°, while the beam wavelength was 0.56300 Å.

Rietveld refinements of XRD data were performed with the software TOPAS version 6 [81]. For cubic and tetragonal CZTS, and monoclinic and cubic CTS, refinements were based on structure models described in previous work from some of the authors [66]. Cubic CZTSe has been modelled as a zinc-blende *F-43m* structure, with full cation disorder on the cation site, keeping an occupancy ratio of 0.5:0.25:0.25 for Cu:Zn:Sn. Tetragonal CZTSe has been modelled with a *I-42m* disordered kesterite structure (see [66] for details). Crystallite size analysis was carried out with the support of macros based on whole powder pattern modelling(WPPM) [70,71]. Microstrain was estimated with built-in TOPAS macros. For the estimate of Debye–Waller coefficients, a unique *B_iso_* has been used for all the cation sites of the tetragonal samples, to allow a comparison with the single cation *B_iso_* of the disordered cubic samples.

**Raman spectroscopy:** Raman spectra were collected with a WiTec spectrometer model alpha 300 RA, using a grating of 1800 g/mm, and with a spectral center at 700 1/cm. A 488 nm laser was employed with a power of 0.5 mW. The measurements were performed with an integration time of 40 s and with 100 accumulations.

**TE measurement:** The Seebeck coefficient and resitivity were simultaneously measured in four contact set-up using LSR-3 Linseis Messgeraete GmbH. The thermal diffusivity (D) was measured using LFA-500 from Linseis Messgeraete GmbH.

**Low temperature mobility measurements:** Resistivity (ρ), magnetoresistivity (ρ(H)- ρ(H=0)/ ρ(H=0), and Hall effect data were measured in a PPMS (Physical Properties Measurement System) by Quantum Design, at temperatures from room temperature down to 10 K and in magnetic fields up to 9 T. More specifically, resistivity was measured using a standard four probe method, and the Hall resistance R_H_ was determined by measuring the transverse resistivity at selected fixed temperatures, sweeping the field from −9 T to 9 T. From ρ and R_H_, the concentration and mobility of charge carriers were extracted in a single band picture.

**Computational:** The ab initio molecular dynamics simulations were performed using the Vienna ab initio simulation package (VASP) [82,83]. A 192-atom and 216-atom supercell were used to represent the ordered and disordered cubic polymorphs of CTS, respectively. While a 64-atom supercell was used to represent both the ordered tetragonal and disordered cubic polymorphs of CZTSe. The disordered structures were modelled by randomly assigning the cation site. The electron-exchange-correlation function was approximated by the PBE [75]. All calculations were performed with an energy cut-off of 400 eV and with a Gaussian charge smearing of 0.1 eV. The irreducible Brillouin zone was sampled on a single point 1 × 1 × 1 Monkhorst Pack gamma-centered k-mesh. Molecular dynamics simulations were performed in a canonical (NVT) ensemble with a Nose-Hoover. In each case, the system was allowed to evolve with a time step of 2 femtoseconds, for 5000 steps, corresponding to a total simulation time of 10 picoseconds. All the structures were relaxed until the force on each atom was <0.01 eV Å^−1^, using a Gaussian smearing of 0.01. The electronic convergence was set to 10^−6^ eV. For the density of states calculation, the hybrid HSE-06 [76] exchange-correlation function was used with a denser k-mesh of 6 × 6 × 6 for disordered cases while 8 × 6 × 8 for monoclinic case. The phonon dispersion curves are obtained using the density functional perturbation theory. PHONOPY code [84] was used to diagonalize the dynamical matrix to calculate the interatomic force constants from which the phonon dispersion curves were calculated.

## Figures and Tables

**Table 1:** Structural information and band gap values for the different polymorphs of CTS, CZTS, and CZTSe. The structures have been produced using VESTA software [65].

CTS	CZTS/CZTSe
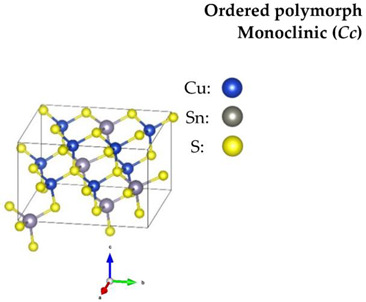	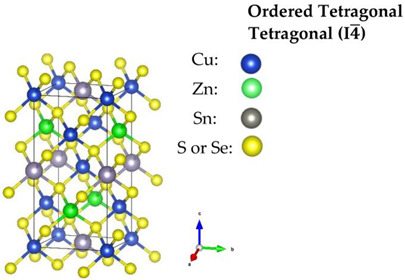
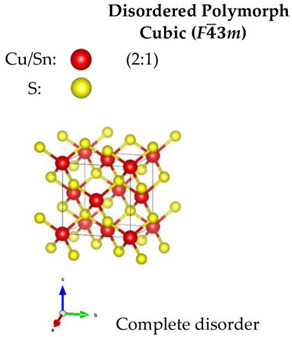	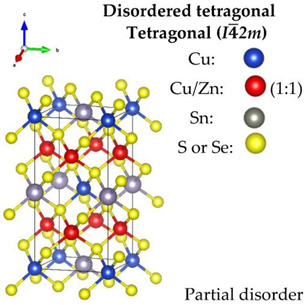
	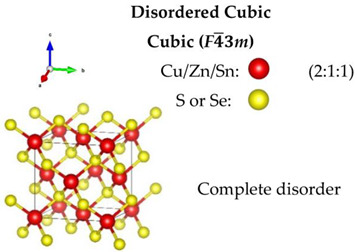

**Table 2:** Structural information and band gap values for the different polymorphs of CTS, CZTS, and CZTSe.

	CTS	CZTS	CZTSe [20]
**Lattice parameters**	*Ordered* a = 6.660(1) Å b = 11.536(2) Å c = 6.660(1) Å	*Tetragonal* [66] a = 5.4345(1) Å b = 5.4345(1) Å c = 10.8380(1) Å	*Tetragonal* a = 5.69706(3) Å b = 5.69706(3) Å c = 11.3472(1) Å
	*Disordered* a = 5.4316(1) Å	*Disordered cubic* [66] a = 5.4150(1) Å	*Disordered cubic* a = 5.68719(9) Å
**Band gap**	*Ordered* [34] 0.99 eV	*Tetragonal* [52] 1.56 eV	*Tetragonal* [67,68,69] 0.9–1.5 eV
	*Disordered* [34] 0.95 eV	*Disordered cubic* [52] 1.53 eV	

## Data Availability

Not applicable.

## Supplementary Materials

The following supporting information can be downloaded at: https://www.mdpi.com/article/10.3390/nano13020366/s1, Figure S1. Temperature dependent SXRD measurement on CTS Ordered (a) and disordered cubic (b) Rietveld refinement performed on ordered and disordered CTS XRD data collected at 350 °C; Figure S2. SAED patterns for of (a) Monoclinic CTS and (b) Disordered Cubic CTS; Figure S3. Magnetoresistivity (ρ(H) − ρ(H = 0))/ρ(H = 0) of (a) Tetragonal CZTS and (b) Disordered Cubic CZTS, measured at T = 50 K; Figure S4. Temperature-dependent mobility measurement of (a) Tetragonal CZTS and (b) Disordered Cubic CZTS; Figure S5. Temperature-dependent electronic part of the thermal conductivity of (a) CTS, (b) CZTS and (c) CZTSe; The relaxed disordered structutre of CTS, CZTS, CZTSe used for calculating electronic properties.
