# Supplementary material for: Mechanochemical Synthesis of Sustainable Ternary and Quaternary Nanostructured Cu2SnS3, Cu2ZnSnS4, and Cu2ZnSnSe4 Chalcogenides for Thermoelectric Applications
Source: Nanomaterials (Basel). 2023 Jan 16;13(2):366. doi: 10.3390/nano13020366 (PMC9866987; doi:10.3390/nano13020366)
Supplement: Supplementary file 1 [file nanomaterials-13-00366-s001.zip › nanomaterials-2139724-supplementary.pdf]

Supporting information: "Mechanochemical Synthesis of Sustainable Ternary and Quaternary Nanostructured Cu_2SnS_3, Cu_2ZnSnS_4, and $Cu_2ZnSnSe_4$ Chalcogenides for Thermoelectric Applications"

Himanshu Nautiyal[1], Ketan Lohani[1], Binayak Mukherjee[1], Eleonora Isotta[1], Marcelo Augusto Malagutti[1], Narges Ataollahi[1], Ilaria Pallecchi[2], Marina Putti[3], Scott T. Misture[4], Luca Rebuffi[5] & Paolo Scardi[1]*

[1] Department of Civil, Environmental and Mechanical Engineering, University of Trento, via Mesiano 77, 38123 Trento, Italy; himanshu.nautiyal@unitn.it (H. N.); ketan.lohani@unitn.it (K. L.); binayak.mukherjee@unitn.it (B. M.); eleonora.isotta@gmail.com (E. I.); marcelo.malagutti@unitn.it (M. A. M.); narges.ataollahi@unitn.it (N. A.);

[2] Consiglio Nazionale delle Ricerche - SuPerconducting and other INnovative materials and devices institute (CNR-SPIN), Department of Physics, via Dodecaneso 33, 16146 Genova, Italy; ilaria.pallecchi@spin.cnr.it (I. P.)

[3] Department of Physics, University of Genova, via Dodecaneso 33, 16146 Genova, Italy; putti@fisica.unige.it (M. P.)

[4] Department of Materials Science & Engineering, Alfred University, Alfred, NY 14802, USA; misture@alfred.edu (S.T.M.)

[5] Argonne National Laboratory, 9700 South Cass Avenue, Lemont, IL 60439 USA; lrebuffi@anl.gov (L. R.)

*Correspondence: paolo.scardi@unitn.it (P.S)

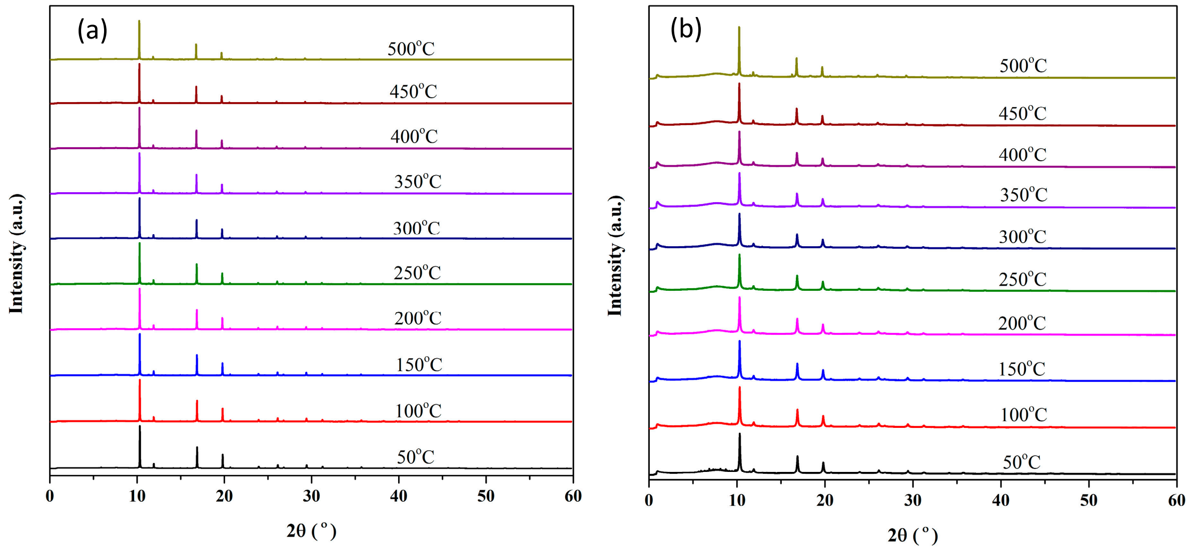

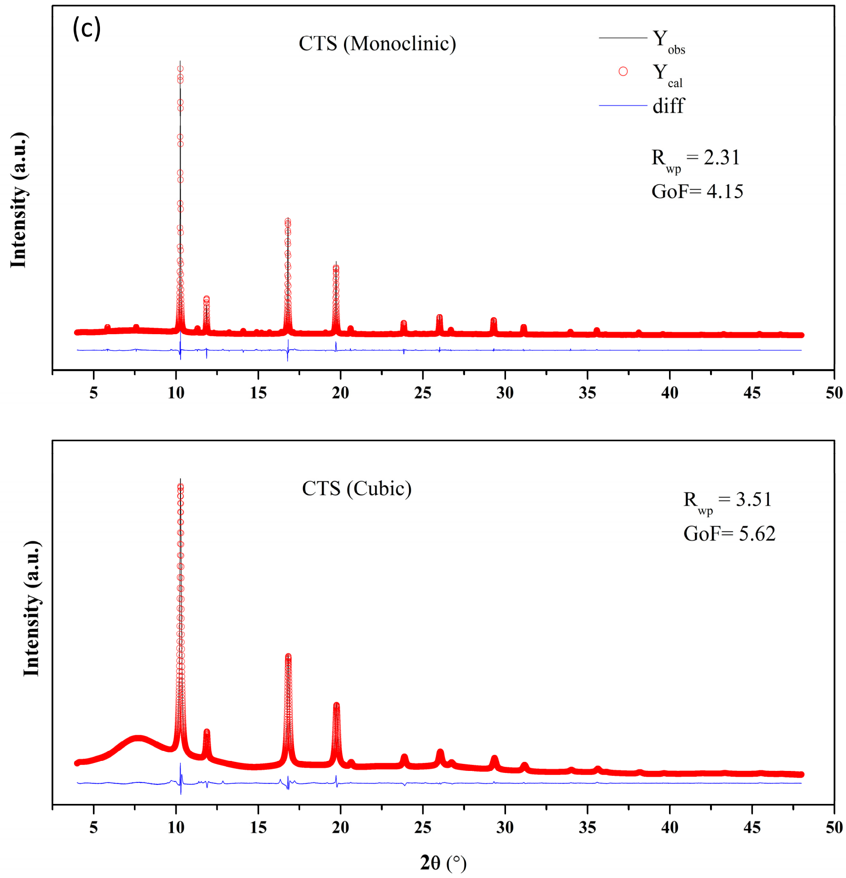

Figure S1. *Temperature dependent SXRD measurement on CTS Ordered (a) and disordered cubic (b) Rietveld refinement performed on ordered and disordered CTS XRD data collected at 350˚C, where* Y_{obs}, Y_{cal}, *and diff are collected, calculated and difference, respectively. The value of* R_{wp} *and GoF for other patterns were similar to the values observed here.*

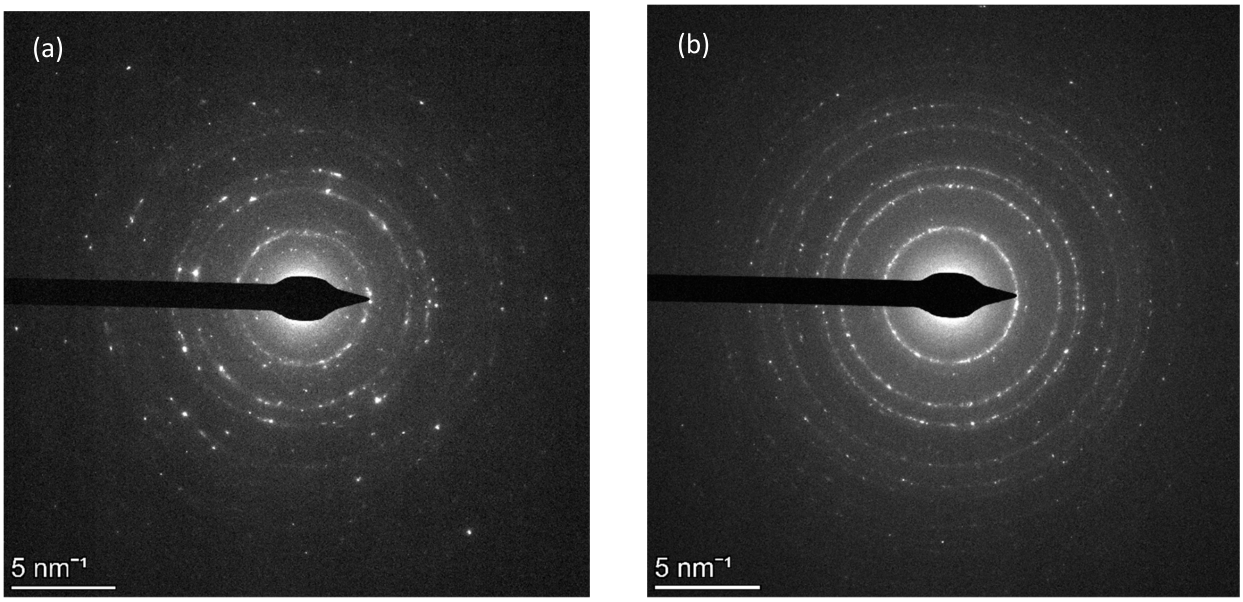

Figure S2. *SAED patterns for of (a)Monoclinic CTS and (b)Disordered Cubic CTS. It is evident from SAED the inner Debye-Scherrer rings are missing for Disordered Cubic CTS representing, (11-1), (021).*

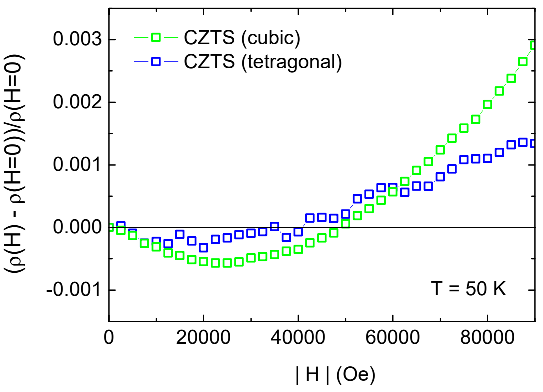

Figure S3. Magnetoresistivity (ρ(H)-ρ(H=0))/ρ(H=0) of (a)Tetragonal CZTS and (b)Disordered Cubic CZTS, measured at T=50K

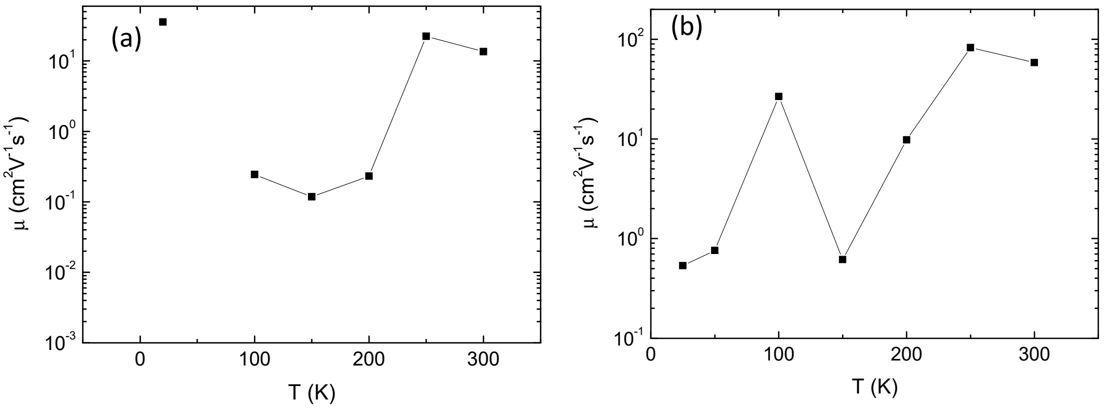

Figure S4. *Temperature-dependent mobility measurement of (a)Tetragonal CZTS and (b)Disordered Cubic CZTS*

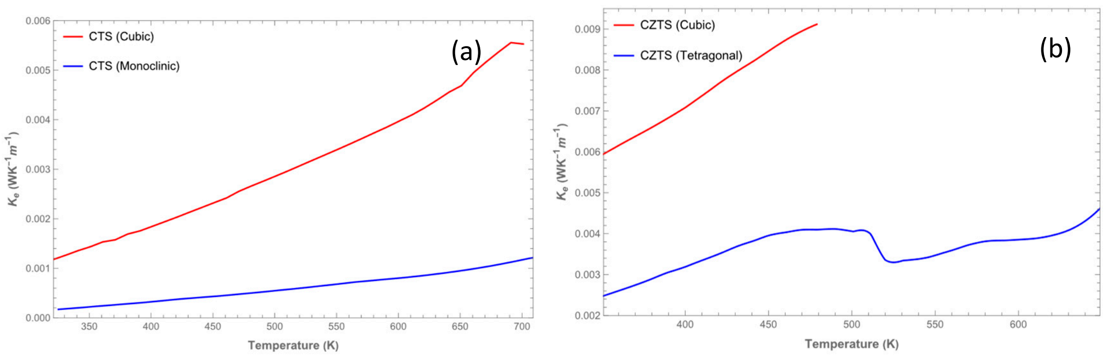

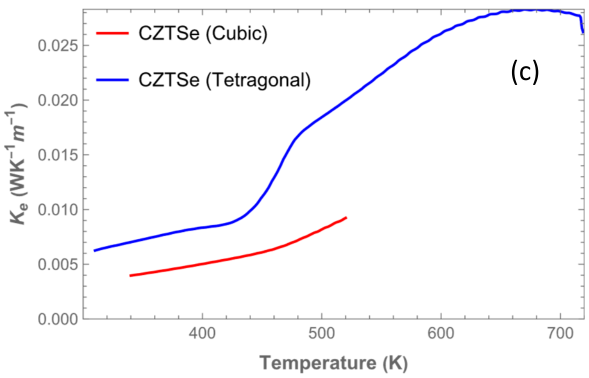

Figure S5. *Temperature-dependent electronic part of the thermal conductivity according to the Wiedemann-Franz law: $k_e = L\,\sigma\,T$ and $L = 1.5 + \exp[-|S|/116]$, where L in 10^{-8} $W\Omega K^{-2}$ and S in μV/K of (a) CTS, (b)CZTS and (c) CZTSe*

The relaxed disordered structutre of CTS, CZTS, CZTSe used for calculating electronic properties:

Disordered CTS-1

1.00000000000000

11.0521121791927346 0.0000000000000000 0.0000000000000000

0.0000000000000000 11.0521121791927346 0.0000000000000000

0.0000000000000000 0.0000000000000000 10.8937928306091436

Cu Sn S

21 11 32

Direct

0.0000000000000000 0.0000000000000000 0.0000000000000000

0.0000000000000000 0.5000000000000000 0.5000000000000000

0.5000000000000000 0.0000000000000000 0.0000000000000000

0.5000000000000000 0.0000000000000000 0.5000000000000000

0.5000000000000000 0.5000000000000000 0.0000000000000000

0.0065877668834062 0.2494657375125300 0.2493503885705550

0.9934122331165938 0.2494657375125300 0.7506495894294432

0.9934122331165938 0.7505342404874682 0.2493503885705550

0.0065877668834062 0.7505342404874682 0.7506495894294432

0.4955536083830125 0.2456471866824401 0.2442584608331799

0.5044463916169875 0.2456471866824401 0.7557415171668183

0.5044463916169875 0.7543527913175581 0.2442584608331799

0.4955536083830125 0.7543527913175581 0.7557415171668183

0.2543528133175599	0.0044463916169875	0.2442584608331799
0.2543528133175599	0.9955536083830125	0.7557415171668183
0.2505342624874700	0.4934122331165938	0.2493503885705550
0.2505342624874700	0.5065877668834062	0.7506495894294432
0.7456471646824383	0.9955536083830125	0.2442584608331799
0.7456471646824383	0.0044463916169875	0.7557415171668183
0.7494657155125282	0.5065877668834062	0.2493503885705550
0.7494657155125282	0.4934122331165938	0.7506495894294432
0.2596844621631647	0.2403155378368353	0.0103606080958940
0.5000000000000000	0.5000000000000000	0.5000000000000000
0.2504241060030381	0.2495758939969619	0.4857781070091676
0.2596844621631647	0.7596844401631628	0.9896393919041060
0.0000000000000000	0.0000000000000000	0.5000000000000000
0.0000000000000000	0.5000000000000000	0.0000000000000000
0.2504241060030381	0.7504240840030363	0.5142218929908324
0.7403155158368335	0.2403155378368353	0.9896393919041060
0.7495758719969601	0.2495758939969619	0.5142218929908324
0.7403155158368335	0.7596844401631628	0.0103606080958940
0.7495758719969601	0.7504240840030363	0.4857781070091676
0.1171186533837769	0.1166593468268360	0.1312780176361912
0.1312117352836921	0.1287421006623646	0.6438808800029676
0.1287428317454484	0.6287428427454458	0.1379182782776454
0.1161747671528701	0.6161747781528675	0.6323817662968025
0.6173330956472540	0.1173330846472496	0.1312581415774261
0.6160926606036625	0.1160926496036652	0.6327858692597914
0.6166593578268333	0.6171186643837743	0.1312780176361912
0.6287421116623619	0.6312117462836895	0.6438808800029676
0.3833406421731667	0.3828813356162257	0.1312780176361912
0.3712578883376381	0.3687882537163105	0.6438808800029676
0.3826669043527531	0.8826669043527460	0.1312581415774261
0.3839073393963375	0.8839073393963375	0.6327858692597914
0.8712571572545542	0.3712571572545542	0.1379182782776454

0.8838252218471325 0.3838252218471325 0.6323817662968025

0.8828813356162257 0.8833406421731667 0.1312780176361912

0.8687882537163105 0.8712578883376381 0.6438808800029676

0.3839073393963375 0.1160926496036652 0.3672141307402086

0.3826669043527531 0.1173330846472496 0.8687418474225765

0.3712578883376381 0.6312117462836895 0.3561191199970324

0.3833406421731667 0.6171186643837743 0.8687219713638115

0.8687882537163105 0.1287421006623646 0.3561191199970324

0.8828813356162257 0.1166593468268360 0.8687219713638115

0.8838252218471325 0.6161747781528675 0.3676182337031975

0.8712571572545542 0.6287428427454458 0.8620817107223573

0.1161747671528701 0.3838252218471325 0.3676182337031975

0.1287428317454484 0.3712571572545542 0.8620817107223573

0.1312117352836921 0.8712578883376381 0.3561191199970324

0.1171186533837769 0.8833406421731667 0.8687219713638115

0.6287421116623619 0.3687882537163105 0.3561191199970324

0.6166593578268333 0.3828813356162257 0.8687219713638115

0.6160926606036625 0.8839073393963375 0.3672141307402086

0.6173330956472540 0.8826669043527460 0.8687418474225765

Disordered CTS -2

1.00000000000000

10.9835867451454678 0.0065491028448356 0.0009814362662756

0.0065526745611678 10.9677768328474130 -0.0024228570139087

0.0010179507687051 -0.0024167454741761 10.8860105502243059

Cu Sn S

22 10 32

Direct

0.7435685556643321 0.2446968819169157 0.0041217695757183

0.9998919269024000 0.5015891140259043 0.5001476759317072

0.5062465711284503 0.0055699404014007 0.0050140821941440

0.4950139957390860 0.9962206345633362 0.4951509148606590

0.4942271211489242	0.4938199366316383	0.0055678920589770
0.0013926521937009	0.2538029530014754	0.2478419194496908
0.9980019083681029	0.2543316382039151	0.7543656271207979
0.9973385364906093	0.7464714348152910	0.2552426669239338
0.0025075019488909	0.7466033898410132	0.7462845783076801
0.4991992832325707	0.2508368137942583	0.2334080524773796
0.5057419011817643	0.5038181883661395	0.4944777228299770
0.5007584605910864	0.7492819490516140	0.2513205525899167
0.4995284272443143	0.7495338404298835	0.7493538408581486
0.2570071424266374	0.0037192426521102	0.2453515811649112
0.2580853298360495	0.0023254150816001	0.7511182526688742
0.2569535020154703	0.4903214802288858	0.2499313945506429
0.2472592967478207	0.5041351798008762	0.7554494719062319
0.7378669910227416	0.0054392287712588	0.2438141926285269
0.7478812274560198	0.0004950961797334	0.7579324356783630
0.7478670212190011	0.4990532027857313	0.2489065154359338
0.7457959735944399	0.4935270057941921	0.7470887842392528
0.2560503752397167	0.2555409260820767	0.0067720006735428
0.4996101638019894	0.2504315662132441	0.7650281738726861
0.2540355290984380	0.2518952797691085	0.4844141181766446
0.2652499745379515	0.7488891378932649	0.0008464834270256
0.0000291381425725	0.9991701937626516	0.5000798875186589
0.0000717630744234	0.4854923534793230	0.0017578989945548
0.2616785485206350	0.7407188177539012	0.5071216034898498
0.9999045122082464	0.0145460210909860	0.0016551760655616
0.7473062707553026	0.2673762214139899	0.4993693327486000
0.7346787374912012	0.7493216427452225	0.0013516293139588
0.7383020139537066	0.7399856226154213	0.4909096000264128
0.1340549978143528	0.1342876465495166	0.1334594827548372
0.1283977725894232	0.1290429554712631	0.6416933756673515
0.1266906056806150	0.6229401041911444	0.1397865745597855
0.1198585857577470	0.6169921388968973	0.6308105492133791

0.6233068411935108	0.1268906134307386	0.1243817171049884
0.6234526772804969	0.1130331443277868	0.6240140296163119
0.6150050178119599	0.6153891464094770	0.1322564577017360
0.6170626107913435	0.6166381286980354	0.6339372971520376
0.3755322690392475	0.3751839949824571	0.1254017788197501
0.3761177748814148	0.3898453710948147	0.6255003188677080
0.3862912070338282	0.8830224974642817	0.1317010762695219
0.3834708743306976	0.8824936693751440	0.6328181542683282
0.8643909983282114	0.3660552903858019	0.1320522057262608
0.8847053594844070	0.3834885248835462	0.6331496802257277
0.8742058504413421	0.8761481037098378	0.1397698437656913
0.8681363054406148	0.8713627812938398	0.6398598132326612
0.3877309200746382	0.1199381826482337	0.3625955398080549
0.3682798294377889	0.1181669688387501	0.8874585363049192
0.3825547827288815	0.6159495339561190	0.3651197760275409
0.3853203408063379	0.6158602564950613	0.8680057321993928
0.8700559519711533	0.1268484996837174	0.3577215714974429
0.8663339261138461	0.1345219236731694	0.8680438627164691
0.8797209669640225	0.6152522962085953	0.3679572399503215
0.8738072761436086	0.6228321685272462	0.8620479995795449
0.1164348424118415	0.3836280050505820	0.3674404183107498
0.1353466619144257	0.3665973924019212	0.8691573410791875
0.1323828641602773	0.8728691627692626	0.3591321559785072
0.1254950046332439	0.8761089223866634	0.8619895774541888
0.6137420140281620	0.3808276160975765	0.3610380543495424
0.6317149213200395	0.3827892605952883	0.8858450534528615
0.6171919591704267	0.8830278492370383	0.3664412963232451
0.6141573732455328	0.8830072371098368	0.8682173982629777

Disordered CZTS

1.0000000000000000

10.9598501947749938 -0.1249688936118855 -0.0250469597288990

-0.1264099280703482 11.0295371370476118 0.0599160245040838

-0.0266992297007424 0.0589943285448715 10.9564733017760236

Cu S Sn Zn

16 32 8 8

Direct

-0.0086094871104244 0.0176019928789748 0.0117549950930625

0.0090637146023007 0.2352502945225316 0.2442983543228030

0.2519913800031896 0.0010761620424628 0.2293020463628186

0.2578110899270293 0.2425376417484607 0.0071856777315485

0.5055444549312837 0.0087513312314706 -0.0050207994653725

0.5031658786725542 0.2588078306070405 0.2335746041921178

0.7450902021245014 0.0105312736137847 0.2441231575854355

0.2749409967710221 0.4825828966669199 0.2545952145691507

0.5041277520780681 0.4934953567697137 0.0059100783374260

0.5017242725523329 0.2506881612351941 0.7596217941554511

0.4987621315182707 0.7384779576387808 0.2431939258425500

0.2567947091579703 0.4943562604149025 0.7489053991486825

0.7089156552464573 0.0298179275070346 0.7951281032453403

0.2613657800872393 0.7365082915164167 0.4881509654337615

0.7279242928526070 0.7592402338527421 0.0065134723735166

0.5023810985469183 0.7520523617004569 0.7527793530047897

0.1292307306114943 0.1248291587797668 0.1231173487096321

0.8582225198463291 0.8882966436471226 0.1177559404151297

0.8715737497316159 0.1317061201648548 0.8848040958879474

0.1205469013176373 0.8864227558364931 0.8854606233643103

0.1257763875853987 0.3706340300176075 0.3601018101993681

0.8660021087242816 0.1367047288531414 0.3652970192603400

0.8732003010380741 0.3605766402607095 0.1330205854985664

0.3744210534627568 0.1281271770065698 0.3576199909437946

0.1574447358951083 0.8666651200625428 0.3610073256659178

0.3909167131778155 0.8825729947828158 0.1291951460811957

0.3769711904926445 0.3718579964504442 0.1194114856581812

0.1394476767835690	0.3644828651341721	0.8714426499303507
0.3790380184359162	0.1310741408101490	0.8820179144386738
0.1224394785759865	0.1257985085804463	0.6341233081329436
0.8315627310028015	0.8931478554775500	0.7079564776667486
0.6188682847438095	0.1270556547749291	0.1270878412754947
0.6091788300531258	0.8738643302634739	0.8848924227246115
0.1410624849829612	0.6100782124299139	0.1261485692940662
0.8590352282496629	0.6324967182632203	0.8874697149428995
0.8756562935134400	0.3797370002543347	0.6261791231226730
0.6213401444473998	0.3725261513847486	0.3725216493473560
0.8908844311307138	0.5941529315359718	0.3560297375382636
0.3663127332994895	0.8663986524331294	0.6315867639800854
0.6085313491483376	0.8837348823027867	0.3570008607563743
0.3793142359457686	0.6208553031333763	0.3677613452099204
0.3879301966280820	0.3796843626157975	0.6354503614079504
0.6200838683394190	0.3783814812618649	0.8769673220591425
0.3888319335521269	0.6107219607061243	0.8659980856147176
0.1500337832483386	0.6094476805265371	0.6119655520338566
0.6255641560485603	0.1392929846418233	0.6350865496934528
0.6195615256023798	0.6242355331932982	0.1235101102146073
0.6203841713771830	0.6285181125685086	0.6281518639558205
-0.0288205276717870	-0.0265704735298441	0.4935267675538384
-0.0038473263255956	0.4826124869901982	-0.0092737099485521
-0.0018111595669480	0.7601962017152303	0.2383377150897734
0.2566351340441114	0.2578276233700614	0.5003305418752404
0.2780977478281365	0.7534045053416784	-0.0115128294749623
0.4975135940376536	0.0035902897243571	0.5065838326826306
0.0019940260451789	0.7468651527155540	0.7337086258694406
0.7671160778170836	0.7690959037888512	0.5003884523093967
-0.0112303529862338	0.2524677667119864	0.7573691952177894
0.2636133762444985	0.0079578860221143	0.7597308878461740
0.7366223715453758	0.2472979067323166	-0.0026134053218011

0.0146711917529684 0.4883593532612543 0.4993240360651845
0.7345323659707420 0.4924903571099992 0.2362560387344842
0.7503463130472496 0.2574086016714243 0.4944872442541364
0.5049232186432423 0.5050213394497090 0.4893310213440047
0.7392817277840106 0.4961200839872347 0.7478692961089842

Disordered CZTSe

1.00000000000000
11.5425481796000007 0.0000000000000000 0.0000000000000000
0.0000000000000000 11.5425481796000007 0.0000000000000000
0.0000000000000000 0.0000000000000000 11.4796619414999999
Zn Cu Sn Se
8 16 8 32
Direct
-0.0091752610058877 0.7364654511814075 0.2609279930909081
0.5030436652531320 0.7755684289782908 0.2142240924459070
0.2406315693714406 0.0140025400958198 0.7340747642683109
0.7885054766415179 0.0197334048102722 0.7767899936972557
0.7504048020837797 0.4731830684685432 0.7626170461669387
-0.0002245688588903 0.5039881058245593 -0.0361886831754705
0.5227258441157406 0.2460077514205972 0.7465011424643688
0.4841748675610680 -0.0031048166907927 0.4995015729188517
-0.0152165144976480 0.2227417128942303 0.2987379338530981
0.2570610931783207 0.5006121507794857 0.7555467864263200
0.2613140059321787 -0.0090551011556622 0.2659546795135315
0.2479312396849483 0.5249042869776248 0.2332651093603103
0.7318171950626275 0.0120786324328238 0.2471355636304772
0.7667298137876398 0.5046240382996998 0.2371607802584911
-0.0217980591018437 0.0082461944331379 0.0142902065939804
0.5090695457999829 0.0138956736322157 -0.0007445266523149
0.4977304945926022 0.4973056392376395 0.0004543864368630
-0.0077295613414229 0.2701858301136066 0.7360211233338299

-0.0253620671837388	0.7537899720249825	0.7624373799370725
0.2523163541776414	0.2311958151946606	0.4991193313895064
0.2585836243564649	0.7638922305674452	0.5010269495949472
0.7406912839842162	0.2561587165074089	0.5119001659852046
0.7711582522641851	0.2284098126654577	0.0200646036788039
0.7687015310744325	0.7343717561223227	0.0070073665852352
0.5088261057762303	0.7472974279166430	0.7788610745420124
0.5122984036092114	0.2437466522128130	0.2461899655012452
0.7350617426823083	0.7524702891129990	0.5126326037785321
0.2425964914893842	0.2538949573864771	0.0019835136465572
0.2474620980582942	0.7490642204326474	0.0187014801705438
0.0181230397741747	-0.0095459373660818	0.4888382269543793
0.0174663944188545	0.4796174739181763	0.5018545877076701
0.4618304827734628	0.5090503974675986	0.4636646238761200
0.1424683170548882	0.1237743577806483	0.3645478678114899
0.1285685118951277	0.6359712367005785	0.3734060897960855
0.6311342992753393	0.1149721647670539	0.3935905108073385
0.6189931488950077	0.6365206768712859	0.3321278140592563
0.3258565080389729	0.3513375848783823	0.3493954210440266
0.3780102704656929	0.8637690386056439	0.3764756182882609
0.8495755038463566	0.3700151038879673	0.3732719308020439
0.8634046658664114	0.8757717028967199	0.3634742837442056
0.1187889603913315	0.4129033025921754	0.1067781832994214
0.1129801444865254	0.8911587331093913	0.1370587888349217
0.6534929625771374	0.3798910503512160	0.1170002148401756
0.6489855656708392	0.8871700681593152	0.0969947627043388
0.3633638934179316	0.1093147371500371	0.1234424864371440
0.3894311119813604	0.6197185898550391	0.1298003401441858
0.8722156659236218	0.1134068975363087	0.1431416824362294
0.8830152253831866	0.6100856279590937	0.1139959980382827
0.3869345104067312	0.3750760054965533	0.8748398319114868
0.3610487545798322	0.9028618101372796	0.8801834537318944

0.8709665226377930	0.3575782188826632	0.8743866416389731
0.8860895187567750	0.8786639781379696	0.8846114034941105
0.1153534125214648	0.1396107434505795	0.8587730281737407
0.1293038158752640	0.6371021835574631	0.8531835023500328
0.6314698606783241	0.1322397113279047	0.8774720773023930
0.6511673094076970	0.6033714081998824	0.8949734283760405
0.3811165640790647	0.1282809903427510	0.6241983121032082
0.3505052672171667	0.6434748361122113	0.6386054717833100
0.8784586830083735	0.1333403448406442	0.6076733255440397
0.8684183237305064	0.5976765933873117	0.6411941652768398
0.1451051871285761	0.3532407360492820	0.6406587656731367
0.1202495124872033	0.8482398922257947	0.6358385759024571
0.6193521917965812	0.3785028612341422	0.6098819198400965
0.6144212662508354	0.9077799396655835	0.6523649729217250
